# Bindarit Inhibits Human Coronary Artery Smooth Muscle Cell Proliferation, Migration and Phenotypic Switching

**DOI:** 10.1371/journal.pone.0047464

**Published:** 2012-10-15

**Authors:** Marcella Maddaluno, Gianluca Grassia, Maria Vittoria Di Lauro, Antonio Parisi, Francesco Maione, Carla Cicala, Daniele De Filippis, Teresa Iuvone, Angelo Guglielmotti, Pasquale Maffia, Nicola Mascolo, Armando Ialenti

**Affiliations:** 1 Department of Experimental Pharmacology, University of Naples Federico II, Naples, Italy; 2 Angelini, ACRAF, S.Palomba-Pomezia, Rome, Italy; 3 Institute of Infection, Immunity and Inflammation, College of Medical, Veterinary and Life Sciences, University of Glasgow, Glasgow, United Kingdom; Harvard Medical School, United States of America

## Abstract

Bindarit, a selective inhibitor of monocyte chemotactic proteins (MCPs) synthesis, reduces neointimal formation in animal models of vascular injury and recently has been shown to inhibit in-stent late loss in a placebo-controlled phase II clinical trial. However, the mechanisms underlying the efficacy of bindarit in controlling neointimal formation/restenosis have not been fully elucidated. Therefore, we investigated the effect of bindarit on human coronary smooth muscle cells activation, drawing attention to the phenotypic modulation process, focusing on contractile proteins expression as well as proliferation and migration. The expression of contractile proteins was evaluated by western blot analysis on cultured human coronary smooth muscle cells stimulated with TNF-α (30 ng/mL) or fetal bovine serum (5%). Bindarit (100–300 µM) reduced the embryonic form of smooth muscle myosin heavy chain while increased smooth muscle α-actin and calponin in both TNF-α- and fetal bovine serum-stimulated cells. These effects were associated with the inhibition of human coronary smooth muscle cell proliferation/migration and both MCP-1 and MCP-3 production. The effect of bindarit on smooth muscle cells phenotypic switching was confirmed *in vivo* in the rat balloon angioplasty model. Bindarit (200 mg/Kg/day) significantly reduced the expression of the embryonic form of smooth muscle myosin heavy chain, and increased smooth muscle α-actin and calponin in the rat carodid arteries subjected to endothelial denudation. Our results demonstrate that bindarit induces the differentiated state of human coronary smooth muscle cells, suggesting a novel underlying mechanisms by which this drug inhibits neointimal formation.

## Introduction

Vascular smooth muscle cell (VSMC) proliferation and migration are key events in intimal hyperplasia occurring in vascular restenosis [1]. After vascular injury, VSMCs exhibit marked differences in morphology, migration, and proliferation rate compared with normal medial cells. Additionally, the highly proliferative VSMCs undergo a shift from a differentiated (contractile) to a dedifferentiated (synthetic, noncontractile) state. This process, called phenotypic modulation, is characterized by the loss of expression of the VSMC-specific genes, such as smooth muscle α-actin (α-SMA) and calponin, as well as a selective upregulation of the embryonic form of smooth muscle myosin heavy chain (SMemb) [2], [3]. The phenotypic switching is accompanied by increased expression of extracellular matrix proteins, cytokines and chemokines [2], [4], [5].

The pro-inflammatory CC chemokine, monocyte chemoattractant protein 1 (MCP-1)/CCL2, plays a pivotal role in intimal hyperplasia via macrophages recruitment and VSMC activation [5], [6]. It has been demonstrated that MCP-1 induces human VSMC proliferation [7], migration [8], and regulates the functional switch of these cells from the contractile to the synthetic phenotype [9].

Bindarit is an anti-inflammatory agent that inhibits MCP-1/CCL2, MCP-3/CCL7 and MCP-2/CCL8 synthesis [10], acting through the down-regulation of NF-kB pathway [11], that shows potent anti-inflammatory activity in animal models of both acute and chronic inflammation [12]–[15]. We have previously demonstrated that oral administration of bindarit inhibits neointimal formation in rodent models of vascular injury by reducing both VSMC proliferation/migration and neointimal macrophage content, effects associated with the inhibition of MCP-1/CCL2 production [16]. Recently, we also demonstrated the efficacy of bindarit on in-stent stenosis in the preclinical porcine coronary stent model [17]. Importantly, a double-blind, randomized, placebo-controlled phase II clinical trial, with the aim of investigating the effect of bindarit in human coronary restenosis, showed that bindarit induced a significant reduction of in-stent late loss [18]. However, the mechanisms underlying the efficacy of bindarit in controlling neointimal formation/restenosis have not been fully elucidated. Therefore, we investigated the effect of bindarit on human coronary VSMC activation, drawing attention to the phenotypic modulation process, focusing on contractile proteins expression as well as proliferation and migration. In addition, we also investigated the effect of bindarit *in vivo* on phenotypic modulation of VSMCs in rat carotid arteries subjected to vascular injury.

**Figure 1:**
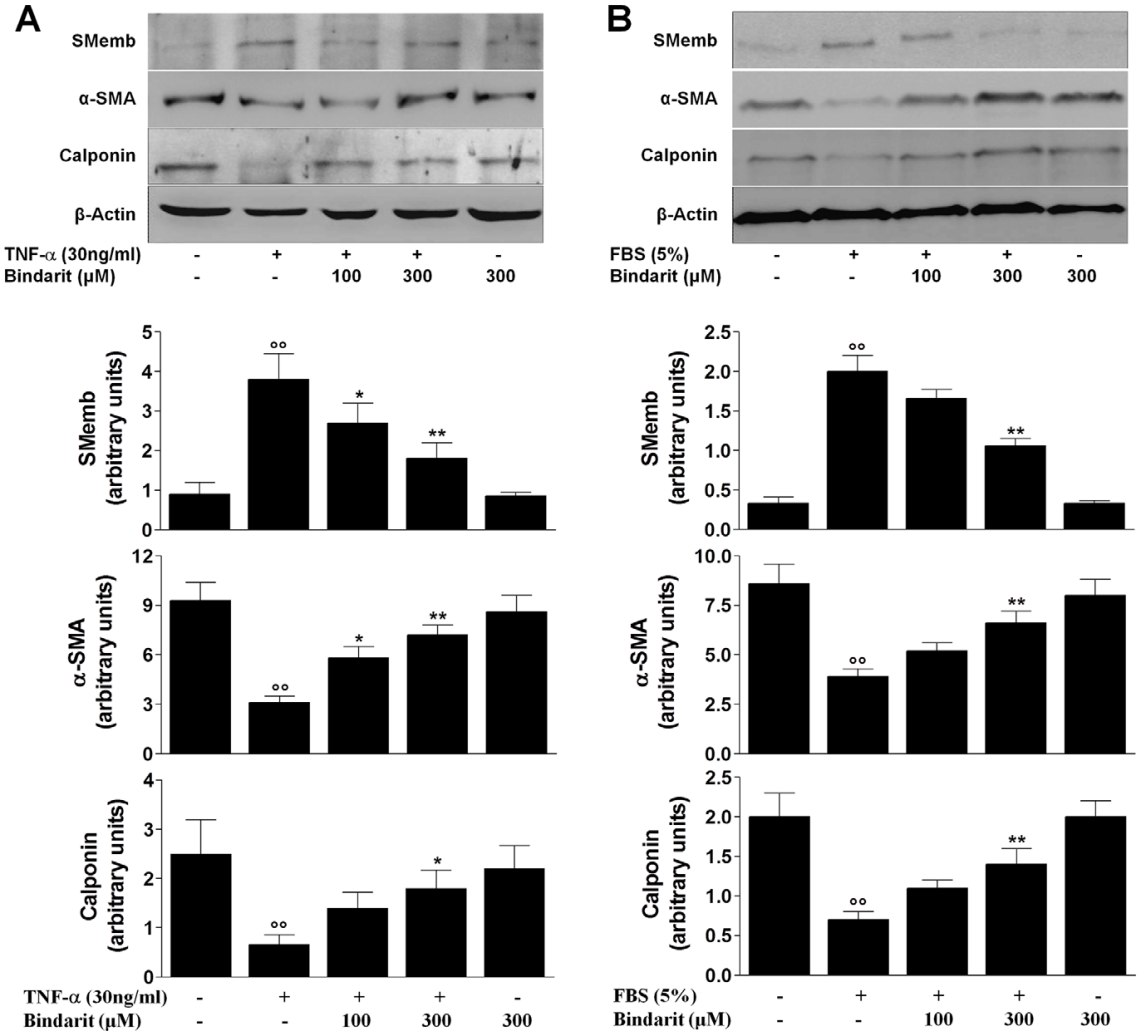
Effect of bindarit on contractile proteins expression in CASMCs. Representative Western blots and relative densitometric analysis showing the effects of bindarit (100 and 300 µM) on contractile proteins expression levels modulated by (**A**) TNF-α (30 ng/mL) or (**B**) FBS (5%). Results are expressed as mean ± SEM of three separate experiments run in triplicate. °°*P*<0.01 *vs* unstimulated cells; *P<0.05, **P<0.01 *vs* untreated cells.

## Methods

### Treatments

Bindarit, 2-methyl-2-[[1-(phenylmethyl)-1H-indazol-3-yl]methoxy] propanoic acid (MW 324.38) was synthesised by Angelini (Angelini Research Center - ACRAF, Italy). Pharmacokinetic studies in rodents show that bindarit is well absorbed when administered by oral route and it has a mean half-life of about 9 h (Product data sheet, Angelini Research Center).

**Figure 2:**
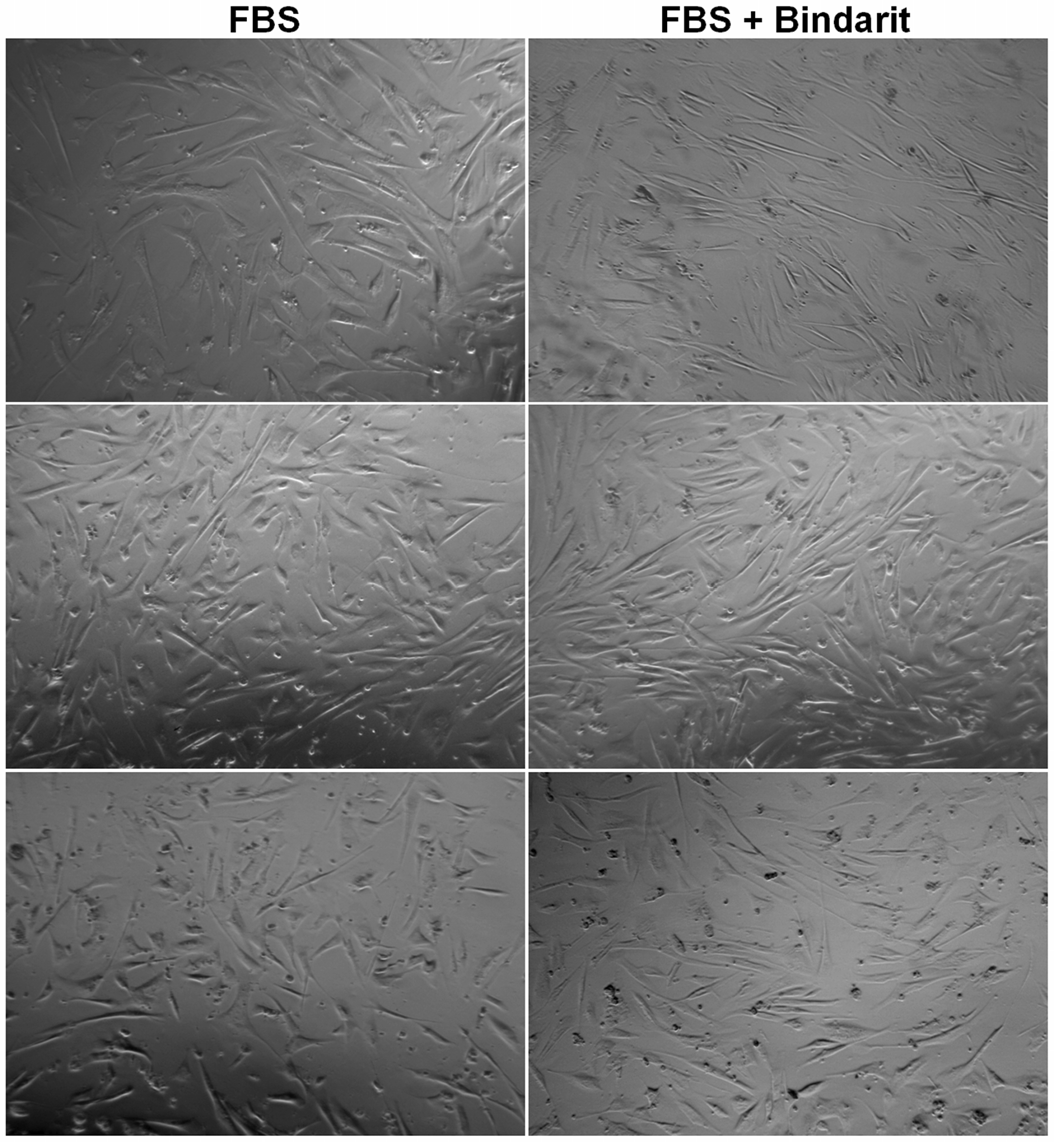
Effect of bindarit on morphological changes induced by FBS in CASMCs. Phase-contrast photomicrographs of CASMCs cultured in medium with 5% FBS for 48 hours with or without bindarit (300 µM).

Animals were treated with bindarit, suspended in 0.5% methylcellulose aqueous solution, at the dose of 100 mg/Kg given orally, by gastric gavage, twice a day [16]. Rats were treated with bindarit from 2 days before angioplasty up to 28 days after. In each experiment control animals received an equal volume of methylcellulose (0.5 mL/100 g). The concentrations of bindarit used for *in vitro* experiments have previously been found to be effective in inhibiting MCP-1 production in rat VSMCs as well as cell proliferation and migration [16].

**Figure 3:**
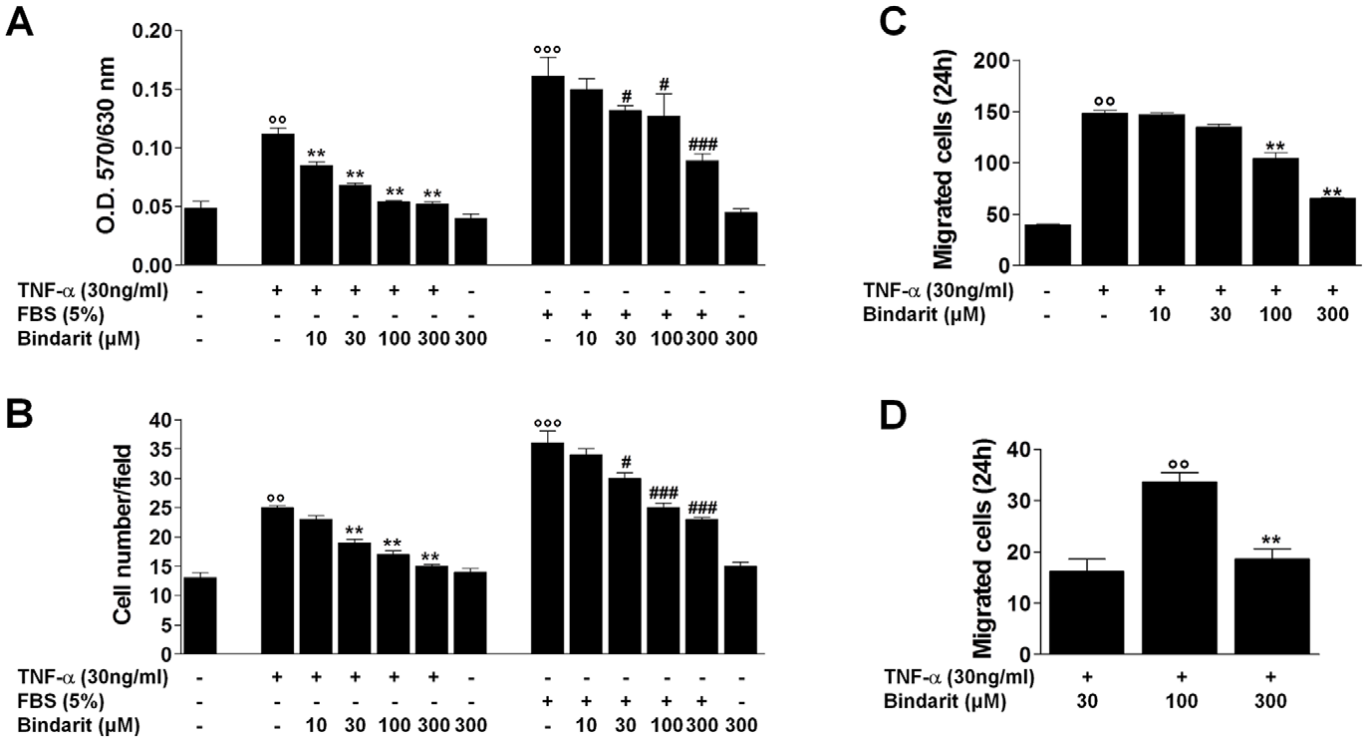
Effect of bindarit on CASMC proliferation, migration and invasion. CASMC proliferation assessed by MTT assay (**A**) and by cell counting expressed as number of cells per field (**B**). Effect of bindarit on CASMC migration (**C**) and invasion (**D**). Results are expressed as mean ± SEM of three separate experiments run in triplicate. **°°** *P*<0.01, **°°°** *P*<0.001 *vs* unstimulated cells; ** *P*<0.01 *vs* TNF-α-stimulated cells; **^#^** *P*<0.05, **^###^** *P*<0.001 *vs* FBS-stimulated cells.

### Cell Culture

Human coronary artery smooth muscle cells (CASMCs) were purchased from Lonza (lot numb: 6F4008 and 16737) [19], grown in Smooth Muscle Basal Medium (SmBM; Lonza) supplemented with 0.5 mg/mL hEGF, 5 mg/mL insulin, 1 mg/mL hFGF, 50 mg/mL gentamicin/amphotericin-B, 5% fetal bovine serum (FBS, Lonza) and used between passages 3–8 for all experiments. Before initiation of the assays, to achieve cell quiescence, CASMCs in exponential growth were switched into SmBM supplemented with 0.1% FBS in the absence of growth factors for 48 hours.

**Figure 4:**
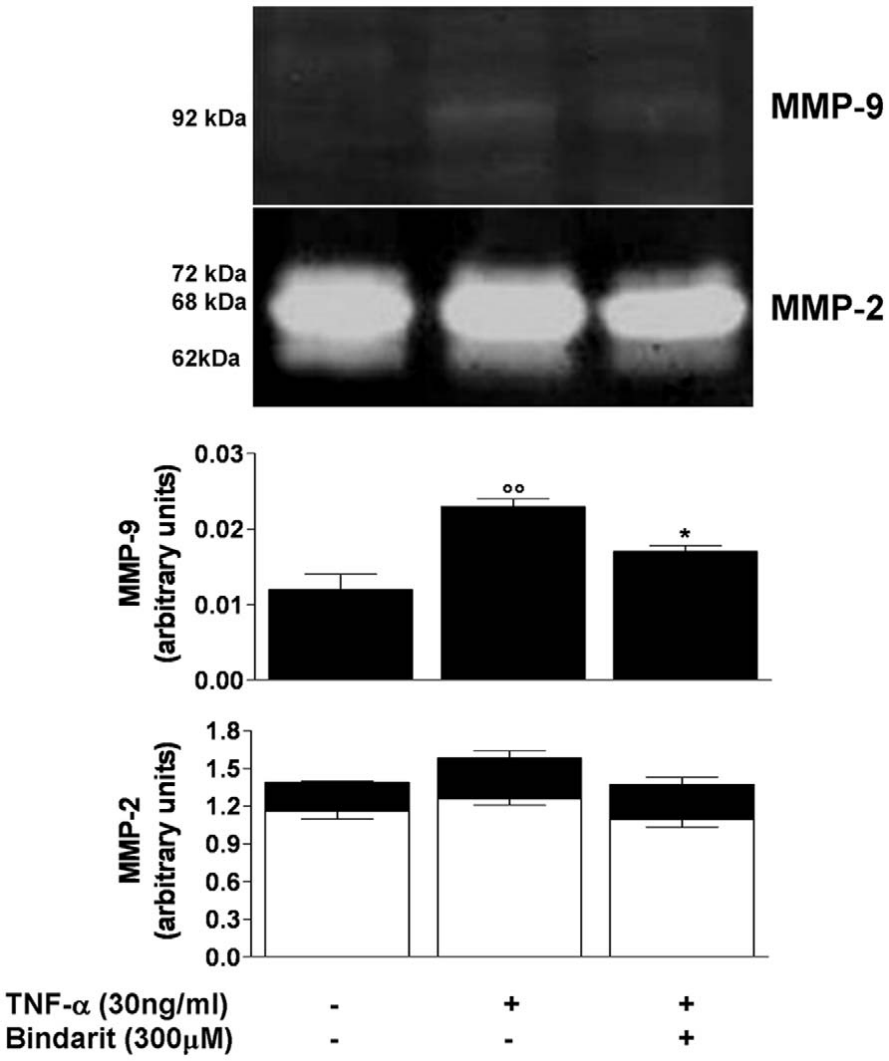
Effect of bindarit on matrix metalloproteinase-2 and matrix metalloproteinase 9 activity. Representative gel zymography of conditioned medium from TNF-α (30 ng/mL)-stimulated CASMCs and relative densitometric analysis showing the effect of bindarit (300 µM) on MMP-9 activated form and both MMP-2 latent (white columns) and activated (black columns) forms. Results are expressed as mean ± SEM of 3 experiments. °°*P*<0.01 *vs* unstimulated cells; °*P*<0.05 *vs* TNF-α-stimulated cells.

### Total Cellular Extracts

CASMCs were cultured in 24 multi-well plates until 90% confluence; after the induction of quiescence, cells were stimulated with tumor necrosis factor-α (TNF-α, 30 ng/mL) or FBS (5%) in presence or absence of bindarit (100–300 µM). After 48 hours cells were washed two times with ice cold PBS and 30 µL/well of lysis buffer (50 mM Tris-HCl, 1% Triton, 1 mM Na_3_VO_4,_ 1 mM EDTA, 0.2 mM PMSF, 25 µg/mL Leupeptin, 10 µg/mL Aprotinin, 10 mM NaF, 150 mM NaCl, 10 mM β-glycerophosphate, 5 mM pyrophosphate, H_2_O) were added. Protein concentration was determined by the Bio-Rad protein assay kit (Bio-Rad).

**Table 1:** Effect of bindarit on MCP-1 production by TNF-α- or FBS-stimulated CASMCs.

	MCP-1 (ng/mL)		
	6 h	12 h	24 h
unstimulated cells	0.1±0.01	0.4±0.04	2.1±0.02
bindarit 300 µM	0.1±0.01	0.4±0.02	2.0±0.03
TNF-α 30 ng/ml	1.6±0.07°°	3.0±1.09°°	13.1±0.12°°
+ bindarit 10 µM	1.5±0.01	2.4±0.04**	11.5±0.15**
+ bindarit 30 µM	1.4±0.01*	2.1±0.02**	10.8±0.10**
+ bindarit 100 µM	1.3±0.02**	1.8±0.05**	10.3±0.29**
+ bindarit 300 µM	1.1±0.06**	1.5±0.05**	8.0±0.05**
FBS 5%	1.5±0.22	5.5±0.26**^+++^**	23.5±1.89**^+++^**
+ bindarit 10 µM	1.5±0.29	4.7±0.61	22.5±1.56
+ bindarit 30 µM	1.4±0.64	3.4±0.75	15.8±2.18**^#^**
+ bindarit 100 µM	1.3±0.74	2.6±0.55**^##^**	10.5±0.87**^###^**
+ bindarit 300 µM	1.0±0.34	2.5±0.30**^##^**	8.8±1.32**^###^**

Results are expressed as mean ± SEM of three separate experiments run in triplicate.

°°*P*<0.01, **^+++^** *P*<0.001 *vs* unstimulated cells; *P<0.05, **P<0.01 *vs* TNF-α-stimulated cells; **^#^** *P*<0.05, **^##^** *P*<0.01, **^###^** *P<*0.001 *vs* FBS-stimulated cells.

**Table 2:** Effect of bindarit on MCP-3 production by TNF-α-stimulated CASMCs.

	MCP-3 (pg/mL)		
	6 h	12 h	24 h
unstimulated cells	70.0±8.00	164.3±40.31	211.3±44.49
bindarit 300 µM	90.3±9.40	178.3±53.35	210.3±52.61
TNF-α 30 ng/ml	116.9±25.71	501.0±78.48°°	714.3±87.83°°°
+ bindarit 10 µM	113.3±16.13	428.0±46.46	671.3±99.47
+ bindarit 30 µM	114.7±18.17	438.0±69.79	477.7±34.80
+ bindarit 100 µM	110.0±20.30	286.0±49.00	440.3±31.84
+ bindarit 300 µM	104.3±21.53	164.7±10.81*	151.3±6.36***

Results are expressed as mean ± SEM of three separate experiments run in triplicate.

°°*P*<0.01, °°°*P*<0.001 *vs* unstimulated cells; *P<0.05, **P<0.01, ***P<0.001 *vs* TNF-α-stimulated cells.

### Western Blot Analysis on CASMCs

CASMCs lysates (20 µg) were separated by Sodium Dodecyl Sulphate - PolyAcrylamide Gel Electrophoresis (SDS-PAGE), transferred onto nitrocellulose membranes (Millipore) and probed with a primary antibody against human α-SMA (1∶5000, Sigma-Aldrich), calponin (1∶5000, Sigma-Aldrich) or MYH9/10 (SMemb, 1∶2000, Santa Cruz). The membranes were washed three times with 0.5% Triton in PBS and incubated with anti-mouse immunoglobulins coupled to peroxidase (1∶1000; DAKO). The immunocomplexes were visualised by the enhanced chemiluminescence (ECL) method, results were analyzed by ImageJ densitometry software and normalized to β-actin.

**Figure 5:**
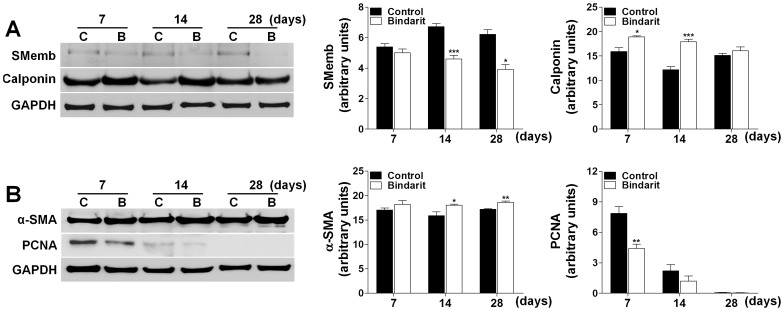
Effect of bindarit on contractile protein expression in rat carotid arteries. A and **B**. Representative Western blots and relative densitometric analysis showing the effect of the oral administration of bindarit (200 mg/Kg/day) on SMemb, calponin, α-SMA and PCNA expression levels in rat carotid arteries at days 7, 14 and 28 days after injury. Results are expressed as mean ± SEM, where n = 4 pools. *P<0.05, **P<0.01 and ***P<0.001 *vs* control group.

### Evaluation of CASMC Morphological Changes

CASMCs were used after the induction of quiescence in 48-well plastic culture plates at the density of 1×10^4^ cells/well. Cells were stimulated with FBS (5%) in presence or absence of bindarit (300 µM). After 48 hours cells were photographed at a magnification of×200 and the images were stored in the image analysis system (LAS, Leica).

**Figure 6:**
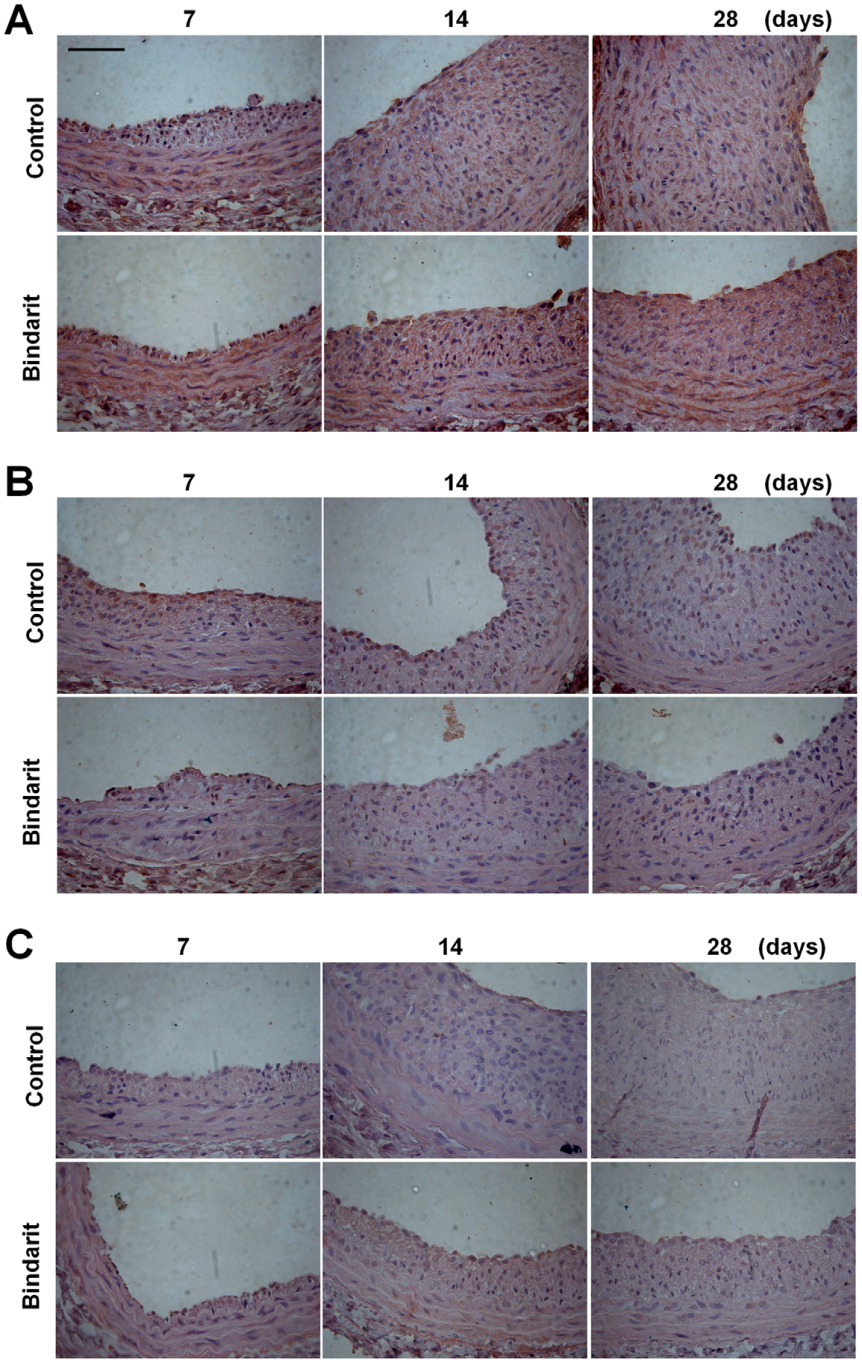
Effect of bindarit on contractile proteins localization in rat carotid arteries. Immohistochemical localization of α-SMA (**A**), SMemb (**B**) and calponin (**C**) expression in rat carotid arteries 7, 14 and 28 days after angioplasty. Bar = 100 µm.

### Cell Proliferation Study

The cell proliferation assay was carried out using the MTT method. CASMCs were plated on 24-well plastic culture plates at the density of 1.5×10^4^ cells/well. After the induction of quiescence, cells were stimulated with TNF-α (30 ng/mL, Provitro) or FBS (5%) for 48 hours in the presence or absence of bindarit (10–300 µM). 0.5 mg/ml of MTT in Phosphate Buffered Saline (PBS) were added and, after 3 hours, a solution containing 50% N,N′-dirnethylformamide and 20% SDS (pH 4.8) was used for the solubilisation of the formazan dye. Absorbance values at 570 nm were determined the next day with an Enzyme-linked immunosorbent assay (ELISA) assay reader (Bio-Rad), using 630 nm as the reference wavelength.

CASMC proliferation was also evaluated as cell duplication by directly counting the cell number. Briefly, 1×10^4^ cells were seeded onto 24-well plastic culture plates and allowed to adhere overnight. After the induction of quiescence, the cells were stimulated with TNF-α (30 ng/mL) or FBS (5%) in presence or absence of bindarit (10–300 µM). After 72 hours, medium was removed, cells were fixed with methanol and stained with 4′,6-diamidino-2-phenylindole (DAPI). Proliferation was evaluated as cell duplication by counting the number of cells in 8 random fields of each well at×100 magnification.

### Chemotactic Migration and Invasion

CASMC migration was evaluated using a modified Boyden chamber (Corning 24 mm Transwell with 8.0 µm pore polycarbonate membrane insert) coated with rat-tail collagen I (Sigma-Aldrich). Biocoat Matrigel invasion chambers (with 8.0 µm pore) were used according to the manufacturer’s instructions for invasion studies (Becton-Dickinson). Briefly, starved CASMCs were trypsinized and pre-treated or not with bindarit (10–300 µM) for 2 hours. Three×10^4^ cells were plated in the upper chamber in 500 µL of 0.1% FBS medium with or without bindarit. The lower chamber was filled with 600 µL of 0.1% FBS medium in the absence (unstimulated cells) or presence of TNF-α (30 ng/mL). After 24 hours the migrated cells were fixed and stained with haematoxylin. Cell migration was quantified by counting the number of cells (magnification×200) per insert.

### Gelatin Zymography

CASMCs were cultured in 96-well culture plates in 10% FBS medium until 90% confluence. After the induction of quiescence, cells were stimulated with TNF-α (30 ng/mL) in the presence or absence of bindarit (300 µM). After 24 hours the media were collected, clarified by centrifugation and subjected to electrophoresis in 8% SDS-PAGE containing 1 mg/mL gelatin. After electrophoresis the gels were re-natured by washing with 2.5% Triton X-100, to remove SDS, and by incubation for 24 h at 37°C in 50 mM Tris buffer containing 200 mM NaCl and 20 mM CaCl_2_, pH 7.4. The gels were stained with 0.5% Coomassie brilliant blue R-250 (Sigma) in 10% acetic acid and 45% methanol and destained with 10% acetic acid and 45% methanol. Bands of gelatinase activity appeared as transparent areas against a blue background. Gelatinase activity was then evaluated by quantitative densitometry.

### Enzyme-linked Immunosorbent Assay (ELISA)

CASMCs were used after the induction of quiescence in 48-well plastic culture plates at the density of 1×10^4^ cells/well. Cells were stimulated with TNF-α (30 ng/mL) in presence or absence of bindarit (10–300 µM). After 6, 12, 24 and 48 hours media were collected, centrifuged at 2000×g for 10 min at 4°C and supernatants were immediately frozen at −80°C until used for MCP-1 (OptEIA, BD) or MCP-3 (Quantikine Human CCL7/MCP-3 Immunoassay, R&D Systems) measurement by ELISA.

### Animals

Male Wistar rats (Harlan Laboratories) weighing 200–300 g were used for the present study. Animals were maintained on a 12/12 h light/dark cycle with free access to food and water at the Department of Experimental Pharmacology, University of Naples Federico II (Permit Number: 064F). All procedures were performed according to Italian ministerial authorization (DL 116/92) and European regulations on the protection of animals used for experimental and other scientific purposes.

### Rat Carotid Balloon Angioplasty

Rats were anaesthetized with an intraperitoneal injection of ketamine (100 mg/Kg) (Gellini International) and xylazine (5 mg/Kg) (Sigma). Endothelial denudation of the left carotid artery was performed by using a balloon embolectomy catheter (2F, Fogarty, Edwards Lifesciences) according to the procedure well validated in our laboratories [20]. Rats were euthanized 7, 14 and 28 days after angioplasty. Carotid arteries were collected and processed as described below.

### Morphometric Analysis

Carotid arteries from rats were fixed by perfusion with phosphate-buffered saline (PBS; pH 7.2) followed by PBS containing 4% formaldehyde through a cannula placed in the left ventricle. Paraffin-embedded sections were cut (6 mm thick) from the approximate middle portion of the artery and stained with haematoxylin and eosin to demarcate cell types. Ten sections from each carotid artery were reviewed and scored under blind conditions. The cross-sectional areas of media and neointima were determined by a computerized analysis system (LAS, Leica). The neointimal and medial areas were computed as follows: neointimal area = internal elastic lamina (IEL) minus lumen area; medial area = external elastic lamina area minus IEL area.

### Total Extracts from Rat Carotid Arteries

Total extracts were prepared from liquid nitrogen frozen pooled carotid arteries (n = 2), crushed into powder, in a mortar with a pestle,and resuspended in 150 µl of lysis buffer (20 mM HEPES, 0.4 mM NaCl, 1.5 mM MgCl2, 1 mM EGTA, 1 mM EDTA, 1% Triton X-100, and 20% glycerol) containing protease inhibitors (1 mM DTT, 0.5 mM PMSF, 15 mg/mL Try-inhibitor, 3 mg/mL pepstatin-A, 2 mg/mL leupeptin, and 40 mM benzamidine) [20], [21]. After centrifugation at 13000×g at 4°C for 30 min, supernatants were collected and stored at −80°C until the assays. Protein concentration was determined by the Bio-Rad protein assay kit (Bio-Rad). MCP-1 levels were quantified by ELISA as described in Supplementary methods in Methods S1.

### Western Blot Analysis on Rat Carotid Arteries

The levels of Proliferating Cell Nuclear Antigen (PCNA), α-SMA, calponin and SMemb were evaluated in total extracts from rat carotid arteries prepared, separated by SDS-PAGE and transferred to nitrocellulose membranes as described above. After incubation with a primary antibody against PCNA (1∶2000, Sigma-Aldrich), α-SMA (1∶5000), calponin (1∶3000) or SMemb (1∶2000), the membranes were washed and incubated with anti-mouse immunoglobulins coupled to peroxidase (1∶2000). The immunocomplexes were visualised by the ECL chemiluminescence method and results were normalized to glyceraldehyde-3-phosphate dehydrogenase (GAPDH).

### Immunohistochemistry

Paraffin sections (6 µm) from rat carotid arteries (7, 14 and 28 days after angioplasty, or naïve animals) were deparaffinised and endogenous peroxidase activity was blocked by incubating with 0.3% H_2_O_2_ following antigenic recovery. The sections were incubated with the primary antibody against α-SMA (1∶100), calponin (1∶50) or SMemb (1∶200) diluted in blocking buffer/0.3% Triton X-100 (MP Biomedicals) in PBS overnight before being washed in TNT wash buffer (Tris–HCl, pH 7.5, 0.15 M NaCl, and 0.05% Tween 20; Sigma). Sections incubated with isotype matched antibodies were used as negative controls. Subsequently, sections were incubated with biotinylated anti-mouse (1∶500, DakoCytomation) diluted in blocking buffer 0.3% Triton X-100, washed in TNT wash buffer, treated with horseradish peroxidise labelled streptavidin, and exposed to diaminobenzidine chromogen with haematoxylin counterstain. The sections were photographed and the images were stored in the image analysis system (LAS, Leica).

### Statistical Analysis

Results are expressed as mean ± SEM of n animals for *in vivo* experiments and mean ± SEM of multiple experiments for in vitro assays. The Student *t* test was used to compare 2 groups or ANOVA (2-tailed probability value) was used with the Dunnett post hoc test for multiple groups using GraphPad Instat 3 software (San Diego, CA). The level of statistical significance was 0.05 per test.

## Results

### Effect of Bindarit on Contractile Proteins Expression in CASMCs

CASMCs were stimulated with TNF-α (30 ng/mL) or FBS (5%) for 48 hours and the lysates from these cells were subjected to Western blot analysis. As shown in Figure 1, bindarit significantly reduced the expression of SMemb in both TNF-α-stimulated cells (by 29% *P*<0.05 and 53% *P*<0.01, at 100 and 300 µM respectively) and FBS-stimulated cells (by 20% *P*<0.01 at 300 µM). The differentiated state of CASMCs induced by bindarit was also confirmed by the significant increased expression of α-SMA in both TNF-α-stimulated cells (by 87% *P*<0.05 and 132% *P*<0.01, at 100 and 300 µM respectively) and FBS-stimulated cells (by 69% *P*<0.01 at 300 µM). Treatment with bindarit at 300 µM also significantly increased calponin expression when compared with both TNF-α-stimulated cells by 172% (*P*<0.05) and FBS-stimulated cells by 100% (*P*<0.01).

### Effect of Bindarit on Morphological Changes Induced by FBS in CASMCs

In addition to VSMC-specific protein expression we examined VSMC morphology. After 48 hours of stimulation with FBS (5%) the CASMCs were characterized by a flattened morphology as result of the dedifferentiation to a synthetic phenotype (Figure 2). Bindarit (300 µM) induced an elongated spindle-shaped phenotype, typical of a differentiated state (Figure 2).

### Effect of Bindarit on CASMC Proliferation

VSMCs plasticity exhibited in response to vascular injury, is characterized by both loss of VSMC-specific proteins expression and the increase in the proliferation.

As shown in Figure 3A, bindarit at 10, 30, 100, and 300 µM significantly (*P*<0.01) inhibited TNF-α (30 ng/mL)-induced CASMC proliferation by 24%, 39%, 52% and 54%, respectively. Similar inhibitory effects of bindarit were observed in FBS (5%)-stimulated CASMCs (Figure 3A).

We also evaluated CASMC proliferation by directly counting the cells (Figure 3B). Bindarit, which was ineffective at 10 µM, significantly (*P*<0.01) inhibited the TNF-α-induced CASMC number increase by 24%, 32% and 40%, at 30, 100, and 300 µM respectively. Similar inhibitory effects of bindarit were observed when FBS was used as stimulant (Figure 3B). Bindarit alone (300 µM) had no effect on cell proliferation/viability (Figure 3A and 3B).

### Effect of Bindarit on CASMC Migration and Invasion

The higher proliferation rate of dedifferentiated VSMCs is accompanied by increased mitogen-mediated migration. Therefore, we evaluated the effect of bindarit (10–300 µM) on TNF-α-induced VSMC chemotaxis. Bindarit significantly (*P*<0.01) inhibited chemotactic migration at 100 and 300 µM by 30% and 55%, respectively (Figure 3C). Moreover, bindarit (300 µM) significantly (*P*<0.01) reduced CASMC invasion by 50% through the Matrigel barrier which mimics extracellular matrix (Figure 3D). Bindarit alone (300 µM) had no effect on both migration and invasion (data not shown).

### Effect of Bindarit on Matrix Metalloproteinase-2 and Matrix Metalloproteinase 9 Activity

Subconfluent cultures of CASMCs were exposed to TNF-α (30 ng/mL) for 24 hours in the presence or absence of bindarit (300 µM) to assess gelatinase production. Gelatin zymography of control supernatants showed the constitutive release of the latent form of matrix metalloproteinase 2 (MMP-2), visualized as a bands at 72 kDa and 68 kDa. Neither the stimulation with TNF-α, nor the treatment with bindarit significantly modified the release of the active form (62 kDa) (Figure 4). The stimulation with TNF-α significantly (*P*<0.01) induced the release of MMP-9 (92 kDa) which was significantly (*P*<0.05) inhibited by bindarit (Figure 4).

### Effect of Bindarit on MCP-1 and MCP-3 Production

The effect of bindarit on MCP-1 and MCP-3 production by CASMCs was determined by ELISA. As shown in table 1, stimulation of CASMCs with TNF-α (30 ng/mL) or FBS (5%) caused a time-dependent increase of MCP-1 levels compared with unstimulated cells. Bindarit (10–300 µM) caused a significant concentration-related inhibition of MCP-1 production. As shown in table 2, bindarit (30–300 µM) significantly reduced MCP-3 production in TNF-α (30 ng/mL) stimulated CASMCs. FBS (5%) had no effect on MCP-3 production (data not shown). Bindarit alone (300 µM) did not significantly affect basal MCP-1 or MCP-3 levels (table 1 and 2).

### Effect of Bindarit on Neointimal Formation in Rat Carotid Arteries

We have previously demonstrated the efficacy of bindarit in reducing balloon-induced neointimal formation in rats, 2 weeks after angioplasty [15]. Here we confirm previously results and extend our observation to the entire time course of neoitimal development, correlating vascular response to injury to contractile protein expression. The oral administration of bindarit significantly (*P*<0.001) inhibited the neointimal growth at 14 and 28 days by 21% and 29% respectively (Supplementary data, Table S1). Similarly, bindarit reduced neointima/media ratio (see Supplementary results in Methods S11 and Table S1). Moreover bindarit significantly (*P*<0.001) induced an increase in lumen area at 14 and 28 days by 26% and 63%, respectively (Supplementary data, Table S1). These effects were associated with a significant reduction of MCP-1 levels in injured carotid arteries of rats treated with bindarit (Supplementary results in Methods S1 and Table S2).

### Effect of Bindarit on Contractile Proteins Expression in Rat Carotid Arteries

As shown in Figure 5A, treatment with bindarit significantly reduced the expression of SMemb at 14 and 28 days (by 31%, *P*<0.001 and 37%*P*<0.05, respectively) and increased the expression of calponin at 7 and 14 days (by 19%, *P*<0.05 and 47%, *P*<0.001). Bindarit also increased the expression of α-SMA at 14 and 28 days (by 13%, *P*<0.05 and 8%, *P*<0.01, respectively) and, as previously demonstrated [16], reduced the expression of PCNA at 7 days (by 44%, *P*<0.01) (Figure 5B).

Localization of contractile proteins in rat carotid arteries was performed by immunohistochemistry to determine the temporal expression and cellular localization. α-SMA resulted highly expressed in the medial VSMCs of non-injured carotid sections (data not shown), while negative control IgG showed no signal (data not shown). At day 7, medial VSMCs, close to the lumen, started to lose α-SMA staining, as consequence of changes in phenotype. At day 14, VSMCs in the media and neointima, although stained with the anti-α-SMA antibody, showed weaker signal than the medial VSMCs at day 7. At day 28, the α-SMA resulted highly expressed in the medial VSMCs, instead the expression in the neointimal cells resulted still weak or absent. Although bindarit did not modify α-SMA localization, it determined a higher α-SMA expression in both media and neointima, at all time points considered (Figure 6A).

Non-injured carotid sections lacked immunoreactive SMemb (data not shown). In contrast, injured carotid arteries showed a remarkable number of cells in the media and neointima strongly positive for SMemb, at all time points considered, while negative control IgG showed no signal (data not shown). The treatment with bindarit reduced the number of the SMemb-positive cells at day 7 and, more interesting, the SMemb-positive cells resulted absent in the media at day 14 and 28 (Figure 6B).

Immunoreactivity for calponin was visible in the medial VSMCs of non-injured carotid sections (data not shown), while negative control IgG showed no signal (data not shown). At all time points considered, the injured arteries lacked immunoreactive calponin. Intriguingly, at day 7 and day 14, the vessels from bindarit-treated rats showed calponin signal in the medial VSMCs (Figure 6C).

## Discussion and Conclusions

VSMC dedifferentiation and phenotype change are thought to be important aspects of vascular wall remodeling during atherosclerosis and neointimal hyperplasia. The present study provides evidence that bindarit induces the differentiated phenotype of VSMCs both *in vitro,* on human coronary VSMCs, and *in vivo,* in the rat carotid balloon angioplasty model. Bindarit differentiation-promoting effect is associated to its ability in suppressing cell proliferation and migration as well as in reducing MCP-1 and MCP-3 production.

In the arterial wall, VSMCs normally exist in a quiescent, differentiated state, representing the contractile phenotype. During neointimal formation VSMCs became activated and change towards the synthetic phenotype characterised by a high rate of proliferation and chemotactic response, changes in the cytoskeleton composition [2] and increased expression of extracellular matrix proteins, cytokines and chemokines [2], [4], [5].

It is well known that chemokines mediate VSMC activation during vascular injury [5], [6], with MCP-1 [7] and MCP-3 [19] shown to directly induce human VSMC proliferation and MCP-1 shown to induce cell migration [8] and the functional switch from the contractile to the synthetic phenotype [9]. This process is characterized by the downregulation of the differentiation markers such as α-SMA and calponin, concurrent with the upregulation of SMemb, that typifies immature VSMCs [2]. Importantly, it is now well established that differentiation and proliferation are not mutually exclusive and that many factors other than VSMC proliferation status influence the differentiation state. Inhibition of proliferation alone is not sufficient to promote VSMC differentiation [22]. However, anti proliferative agents used for inhibition of experimental neointimal formation, like simvastatin [23], or human restenosis, like rapamycin [24], are also able to induce VSMC differentiated phenotype [24], [25].

Bindarit is a selective inhibitor of MCP-1/CCL2, MCP-3/CCL7, and MCP-2/CCL8 synthesis [10] acting through the down-regulation of NF-kB pathway [11]. It is effective in reducing neointimal formation in both non-hyperlipidemic and hyperlipidemic rodent models of vascular injury [16] as well as in a model of coronary in-stent stenosis in the pig [17] having a direct effect on VSMC proliferation/migration and reducing neointimal macrophage content [16], [17]. Recently, a phase II clinical trial, has demonstrated the efficacy of bindarit in reducing in-stent late loss [18]. To better understand the effect of bindarit on human VSMC, here we evaluated the phenotypic modulation of CASMC analyzing the contractile proteins (α-SMA, calponin and SMemb) expression. α-SMA is known to be expressed in a wide variety of non-VSMC cell types, under certain circumstances, for this reason we also analyzed calponin, that is univocally expressed by fully differentiated, mature VSMC [2]. We observed that the expression of contractile proteins in CASMCs changed in response to stimulation with FBS and the proinflammatory cytokine TNF-α, with a reduction of α-SMA and calponin, and a concomitant increase of SMemb. These changes were significantly reversed by bindarit. CASMCs grown in presence of FBS exhibited a flattened morphology, feature of the synthetic phenotype. After bindarit treatment cells acquired the elongated and spindle-shaped morphology, typical feature of the contractile phenotype. Further bindarit inhibited CASMC proliferation, migration and invasion through the Matrigel barrier and reduced metalloproteinase (MMP)-9 activity, which is known to be key for VSMC migration into the intimal area [26], [27]. Bindarit also reduced the levels of both MCP-1 and MCP-3, data in line with results observed in other species [16], [17].

The effect of bindarit on VSMC phenotypic switching was confirmed *in vivo* in the rat carotid arteries subjected to balloon-induced endothelial denudation, an ideal experimental model for studying VSMC behaviour [7]. The inhibition of neointimal formation observed in bindarit treated rats was associated with a modulation of the contractile proteins expression patterns. Indeed, treatment with bindarit reduced the expression of SMemb and increased the expression of α-SMA and calponin after vascular injury.

In conclusion, our study demonstrates that bindarit regulates the contractile proteins expression and phenotype switching of VSMCs. Our data suggest a novel underlying mechanisms by which bindarit can inhibit neointimal formation in human restenosis.

## Supporting Information

Table S1 Morphometric analysis of rat carotid arteries 7, 14 and, 28 days after angioplasty. The results are expressed as mean ± SEM (n = 10). *P<0.05, ***P<0.001 *vs* control group.
(DOC)

Table S2 MCP-1 levels in injured carotid arteries. The results are expressed as mean ± SEM (n = 4). *P<0.05, **P<0.01 *vs* control group.
(DOC)

Methods S1 (DOC)

## References

[1] Marx SO, Totary-Jain H, Marks AR (2011) Vascular smooth muscle cell proliferation in restenosis. Circ Cardiovasc Interv 4: 104–111. 21325199 10.1161/CIRCINTERVENTIONS.110.957332 PMC3816546

[2] Owens GK, Kumar MS, Wamhoff BR (2004) Molecular regulation of vascular smooth muscle cell differentiation in development and disease. Physiol Rev 84: 767–801. 15269336 10.1152/physrev.00041.2003

[3] Regan CP, Adam PJ, Madsen CS, Owens GK (2000) Molecular mechanisms of decreased smooth muscle differentiation marker expression after balloon injury. J Clin Invest 106: 1139–1147. 11067866 10.1172/JCI10522 PMC301419

[4] Charey DJ (1991) Control of growth and differentiation of vascular cells by extracellular matrix proteins. Annu Rev Physiol 53: 161–177. 2042957 10.1146/annurev.ph.53.030191.001113

[5] Schober A (2008) Chemokines in vascular dysfunction and remodeling. Arterioscler Thromb Vasc Biol 28: 1950–1959. 18818421 10.1161/ATVBAHA.107.161224

[6] Schober A, Zernecke A, Liehn EA, von Hundelshausen P, Knarren S, Kuziel WA, et al (2004) Crucial role of the CCL2/CCR2 axis in neointimal hyperplasia after arterial injury in hyperlipidemic mice involves early monocyte recruitment and CCL2 presentation on platelets. Circ Res. 95: 1125–33. 10.1161/01.RES.0000149518.86865.3e 15528472

[7] Selzman CH, Miller SA, Zimmerman MA, Gamboni-Robertson F, Harken AH, et al (2002) Monocyte chemotactic protein-1 directly induces human vascular smooth muscle proliferation. Am J Physiol Heart Circ Physiol 283: H1455–H1461. 12234797 10.1152/ajpheart.00188.2002

[8] Parenti A, Bellik L, Brogelli L, Filippi S, Ledda F (2004) Endogenous VEGF-A is responsible for mitogenic effects of MCP-1 on vascular smooth muscle cells. Am J Physiol Heart Circ Physiol. 286: H1978–84. 10.1152/ajpheart.00414.2003 14693680

[9] Denger S, Jahn L, Wende P, Watson L, Gerber SH, et al (1999) Expression of monocyte chemoattractant protein-1 cDNA in vascular smooth muscle cells: induction of the synthetic phenotype: a possible clue to VSMC differentiation in the process of atherogenesis. Atherosclerosis 144: 15–23. 10381273 10.1016/s0021-9150(99)00033-7

[10] Mirolo M, Fabbri M, Sironi M, Vecchi A, Guglielmotti A, et al (2008) Impact of the anti-inflammatory agent bindarit on the chemokinome: selective inhibition of the monocyte chemotactic proteins. Eur Cytokine Netw 19: 119–22. 18775807 10.1684/ecn.2008.0133

[11] Mora E, Guglielmotti A, Biondi G, Sassone-Corsi P (2012) Bindarit: an anti-inflammatory small molecule that modulates the NFκB pathway. Cell Cycle 11: 159–69. 22189654 10.4161/cc.11.1.18559 PMC3356824

[12] Perico N, Benigni A, Remuzzi G (2008) Present and future drug treatments for chronic kidney diseases: evolving targets in renoprotection. Nat Rev Drug Discov 7: 936–853. 18846102 10.1038/nrd2685

[13] Rulli NE, Guglielmotti A, Mangano G, Rolph MS, Apicella C, et al (2009) Amelioration of alphavirus-induced arthritis and myositis in a mouse model by treatment with bindarit, an inhibitor of monocyte chemotactic proteins. Arthritis Rheum 60: 2513–2523. 19644852 10.1002/art.24682

[14] Bhatia M, Devi Ramnath RD, Chevali L, Guglielmotti A (2005) Treatment with bindarit, a blocker of MCP-1 synthesis, protects mice against acute pancreatitis. Am JPhysiol Gastrointest Liver Physiol 288: G1259–G1265. 15691869 10.1152/ajpgi.00435.2004

[15] Bhatia M, Landolfi C, Basta F, Bovi G, Ramnath RD, et al (2008) Treatment with bindarit, an inhibitor of MCP-1 synthesis, protects mice against trinitrobenzene sulfonic acid-induced colitis. Inflamm Res 57: 464–471. 18827968 10.1007/s00011-008-7210-y

[16] Grassia G, Maddaluno M, Guglielmotti A, Mangano G, Biondi G, et al (2009) The anti-inflammatory agent bindarit inhibits neointima formation in both rats and hyperlipidaemic mice. Cardiovasc Res 84: 485–93. 19592568 10.1093/cvr/cvp238 PMC2777949

[17] Ialenti A, Grassia G, Gordon P, Maddaluno M, Di Lauro MV, et al (2011) Inhibition of in-stent stenosis by oral administration of bindarit in porcine coronary arteries. Arterioscler Thromb Vasc Biol 31: 2448–54. 21852559 10.1161/ATVBAHA.111.230078

[18] Colombo A, Limbruno U, Lettieri C, Lioy E, Guglielmotti A, et al (2012) A double blind randomized study to evaluate the efficacy of bindarit in preventing coronary stent restenosis. J Am Coll Cardiol. 59: E11–E11. 10.4244/EIJY15M12_03 26690313

[19] Maddaluno M, DiLauro MV, Di Pascale A, Santamaria R, Guglielmotti A, et al (2011) Monocyte chemotactic protein-3 induces human coronary smooth muscle cell proliferation. Atherosclerosis 217: 113–9. 21536288 10.1016/j.atherosclerosis.2011.04.002

[20] Grassia G, Maddaluno M, Musilli C, De Stefano D, Carnuccio R, et al (2010) The IκB kinase inhibitor nuclear factor-κB essential modulator-binding domain peptide for inhibition of injury-induced neointimal formation. Arterioscler Thromb Vasc Biol 30: 2458–66. 20930169 10.1161/ATVBAHA.110.215467

[21] Maffia P, Grassia G, Di Meglio P, Carnuccio R, Berrino L, et al (2006) Neutralization of Interleukin-18 Inhibits Neointimal Formation in a Rat Model of Vascular Injury. Circulation 114: 430–437. 16864728 10.1161/CIRCULATIONAHA.105.602714

[22] Alexander MR, Owens GK (2012) Epigenetic control of smooth muscle cell differentiation and phenotypic switching in vascular development and disease. Annu Rev Physiol. 74: 13–40. 10.1146/annurev-physiol-012110-142315 22017177

[23] Indolfi C, Cioppa A, Stabile E, Di Lorenzo E, Esposito G, et al (2000) Effects of hydroxymethylglutaryl coenzyme a reductase inhibitor simvastatin on smooth muscle cell proliferation in vitro and neointimal formation in vivo after vascular injury. J Am Coll Cardiol 35: 214–21. 10636283 10.1016/s0735-1097(99)00526-4

[24] Martin KA, Merenick BL, Ding M, Fetalvero KM, Rzucidlo EM, et al (2007) Rapamycin promotes vascular smooth muscle cell differentiation through insulin receptor substrate-1/phosphatidylinositol 3-kinase/akt2 feedback signaling. J Biol Chem 282: 36112–36120. 17908691 10.1074/jbc.M703914200

[25] Wada H, Abe M, Ono K, Morimoto T, Kawamura T, et al (2008) Statins activate GATA-6 and induce differentiated vascular smooth muscle cells. Biochem Biophys Res Commun 374: 731–736. 18671946 10.1016/j.bbrc.2008.07.098

[26] Newby AC, Zaltsman AB (2000) Molecular mechanisms in intimal hyperplasia. J Pathol 190: 300–309. 10685064 10.1002/(SICI)1096-9896(200002)190:3<300::AID-PATH596>3.0.CO;2-I

[27] Bendeck MP, Zempo N, Clowes AW, Galardy RE, Reidy MA (1994) Smooth muscle cell migration and matrix metalloproteinase expression after arterial injury in the rat. Circ Res 75: 539–545. 8062427 10.1161/01.res.75.3.539

