# Supplementary material for: Bindarit Inhibits Human Coronary Artery Smooth Muscle Cell Proliferation, Migration and Phenotypic Switching
Source: PLoS One. 2012 Oct 15;7(10):e47464. doi: 10.1371/journal.pone.0047464 (PMC3471825; doi:10.1371/journal.pone.0047464)
Supplement: Table S1 — Morphometric analysis of rat carotid arteries 7, 14 and, 28 days after angioplasty. The results are expressed as mean ± SEM (n = 10). *P<0.05, ***P<0.001 vs control group. (DOC) [file pone.0047464.s001.doc]

**Table S1**.

|  | **7 Days** | | **14 Days** | | **28 Days** | |
| --- | --- | --- | --- | --- | --- | --- |
|  | **Control** | **Bindarit** | **Control** | **Bindarit** | **Control** | **Bindarit** |
| Vessel area (mm^2^) | 0.662±0.035 | 0.688±0.020 | 0.715±0.036 | 0.727±0.028 | 0.647±0.028 | 0.684±0.030 |
| Media area (mm^2^) | 0.161±0.010 | 0.167±0.014 | 0.179±0.014 | 0.167±0.012 | 0.176±0.013 | 0.168±0.008 |
| Lumen area (mm^2^) | 0.430±0.024 | 0.462±0.020 | 0.291±0.013 | 0.367±0.017*** | 0.196±0.011 | 0.319±0.018*** |
| Neointimal area (mm^2^) | 0.070±0.010 | 0.058±0.012 | 0.244±0.013 | 0.192±0.010*** | 0.275±0.014 | 0.196±0.011*** |
| Neointima/Media Ratio | 0.441±0.050 | 0.371±0.070 | 1.398±0.064 | 1.178±0.001* | 1.612±0.097 | 1.191±0.070*** |
