# Supplementary material for: Bindarit Inhibits Human Coronary Artery Smooth Muscle Cell Proliferation, Migration and Phenotypic Switching
Source: PLoS One. 2012 Oct 15;7(10):e47464. doi: 10.1371/journal.pone.0047464 (PMC3471825; doi:10.1371/journal.pone.0047464)
Supplement: Table S2 — MCP-1 levels in injured carotid arteries. The results are expressed as mean ± SEM (n = 4). *P<0.05, **P<0.01 vs control group. (DOC) [file pone.0047464.s002.doc]

**Table S2**.

|  | **MCP-1 (ng/mg)** | | |
| --- | --- | --- | --- |
|  | **7 days** | **14 days** | **28 days** |
| **Control** | 12.59±0.55 | 19.03±0.76 | 26.01±1.29 |
| **Bindarit** | 10.00±0.32** | 13.00±0.79** | 15.94±2.94* |
