# Supplementary material for: Bindarit Inhibits Human Coronary Artery Smooth Muscle Cell Proliferation, Migration and Phenotypic Switching
Source: PLoS One. 2012 Oct 15;7(10):e47464. doi: 10.1371/journal.pone.0047464 (PMC3471825; doi:10.1371/journal.pone.0047464)
Supplement: Methods S1 — (DOC) [file pone.0047464.s003.doc]

**Supplementary methods**

**Enzyme-linked immunosorbent assay (ELISA) on rat carotid arteries**

Total extracts were prepared as described in Method section (Total extracts from rat carotid arteries). MCP-1 levels were quantified using the ELISA kit (OptEIATM, Biosciences) according to the manufacturer’s instructions. All measurements were performed in duplicate. The values were normalized by protein concentrations measured by the Bio-Rad protein assay kit (Bio-Rad).

**Supplementary results**

**Effect of bindarit on morphological measurement**

Bindarit induced the significant reduction of N/M ratio 14 days (*P*<0.05) and 28 days (*P*<0.001) after injury (table S1). No effects of bindarit were observed on media and vessel area (table S1).

**Effect of bindarit on MCP-1 production in rat carotid arteries**

A significant time-dependent increase in MCP-1 production in the injured arteries at 7, 14 and, 28 days after injury was observed (Table S2). Bindarit was able to inhibit the MCP-1 protein expression throughout the time course considered, by 21% (*P*<0.01; n=4), 32% (*P*<0.01; n=4), and 39% (*P*<0.05; n=4) at 7, 14, and 28 days after injury, respectively.
